# Three-Month Feeding Integration With *Bifidobacterium* Strains Prevents Gastrointestinal Symptoms in Healthy Newborns

**DOI:** 10.3389/fnut.2018.00039

**Published:** 2018-05-25

**Authors:** Irene Aloisio, Flavia Prodam, Enza Giglione, Nicole Bozzi Cionci, Arianna Solito, Simonetta Bellone, Loredana Baffoni, Luca Mogna, Marco Pane, Gianni Bona, Diana Di Gioia

**Affiliations:** ^1^Department of Agricultural and Food Sciences, University of Bologna, Bologna, Italy; ^2^Department of Health Sciences, Università degli Studi del Piemonte Orientale, Novara, Italy; ^3^Interdisciplinary Research Center of Autoimmune Diseases (IRCAD), Università degli Studi del Piemonte Orientale, Novara, Italy; ^4^Biolab Reserach, Novara, Italy

**Keywords:** probiotic, *Bifidobacterium breve*, infant colic, functional gastrointestinal disorders, breastfeeding, bottle-feeding, microbiota

## Abstract

Infantile functional gastrointestinal disorders are common in the first months of life. Their pathogenesis remains unknown although evidences suggest multiple independent causes, including gut microbiota modifications. Feeding type, influencing the composition of intestinal microbiota, could play a significant role in the pathogenesis. Previous studies supported probiotic supplementation success against colics, however mainly *Lactobacillus* spp. were tested. The aim of this study was to evaluate the effectiveness against functional gastrointestinal disorders of a *Bifidobacterium breve* based probiotic formulation including in the study both breast-fed and bottle-fed subjects. Two hundred and sixty-eight newborns were enrolled within 15 days from birth. One hundred and fifty-five of them effectively entered the study and were randomized in probiotic and placebo group, receiving the formulation for 90 days. The probiotic formulation consists of a 1:1 mixture of 2 strains of *B. breve* prepared in an oily suspension and administered in a daily dosage of 5 drops containing 10^8^ CFU of each strain. Absolute quantification of selected microbial groups in the faeces was performed using qPCR. Anthropometric data, daily diary minutes of crying, number of regurgitations, vomits and evacuations, and colour and consistency of stools were evaluated before and after treatment. The study confirmed the positive role of breast milk in influencing the counts of target microbial groups, in particular the bifidobacteria community. No adverse events upon probiotic administration were reported, suggesting the safety of the product in this regimen. *B. breve* counts increased significantly in all administered newborns (*p* < 0.02). The study demonstrates that a 3 months treatment with *B. breve* strains in healthy breast-fed newborns helps to prevent functional gastrointestinal disorders, in particular reducing 56% of daily vomit frequency (*p* < 0.03), decreasing 46.5% of daily evacuation over time (*p* < 0.03), and improving the stool consistency (type 6 at the Bristol Stool chart instead of type 5) in those at term (*p* < 0.0001). Moreover, a significant reduction (8.65 vs. 7.98 LogCFU/g of feces, *p* < 0.03) of *B. fragilis* in the bottle-fed group receiving the probiotic formulation was observed.

## Introduction

Infant colic is a common disorder in the first 3 months of childhood that affects up to 30% of newborns and is characterized by paroxysmal, excessive, incontrollable crying without identifiable causes (1). Wessel et al. (2) gave the first definition of this disorder as a condition of crying or fussing that lasts more than 3 h per day, more than 3 days per week. For a clinical purpose, the Rome IV consensus group (3) recently revised the diagnostic criteria including the age of newborn (<5 months), giving less importance to the amount of crying considering instead the prolonged and unsoothable character of the crying episodes as well as irritability that cannot be prevented or resolved by caregivers. Symptoms, such as flushing of the face, meteorism, thighs flexion and flatulence, begin in the second week of life, in both breast-fed and formula-fed infants, and usually resolve spontaneously over time (4).

Infant colic represents a serious problem for the family, because caregivers have difficulties in dealing with these incontrollable crises often resulting in stress and concerns; a prospective European multicenter study carried out by Vik et al. (5) revealed that infantile colic and prolonged crying are associated with high maternal depression scores. Similarly, regurgitation, vomit and constipation frequently require a pediatrician visit during the first 6 months of life and are often responsible for feeding changes, and use of medical treatments (6, 7). Moreover, several consequences were associated to the presence of colics in the early stage of life: children with a history of colics have a higher prevalence of functional gastrointestinal disorders later in life (8) and children with migraine were more likely to have experienced infantile colic than those without migraine (7). Therefore, an effective preventive strategy against functional gastrointestinal disorders is envisaged.

Despite 40 years of research, the etiology of colic crises and other functional gastrointestinal disorders has not been fully clarified. It has been suggested that a number of behavioral factors (psychological and social), nutritional factors (food hypersensitivity or allergy), intestinal dysmotility and low grade intestinal inflammation can contribute to its occurrence (6, 9). Being a typical disorder of the gastrointestinal tract, it is not surprising that imbalance in the gut microbiota composition has been suggested to play a role in the pathogenesis of these conditions. The gut microbiota has a very close relation with the host contributing to the normal human physiology: it can provide a barrier for colonization of pathogens, synthesize vitamins, and other beneficial compounds and stimulate the immune system (10). The neonatal period is a crucial stage for gastrointestinal colonization, a balanced composition of the gut microbiota resulting in a positive effects on the host health (11). Colicky infants have a reduced fecal-bacterial diversity and stability, compared to the healthy ones. They also show a higher prevalence of gram negative bacteria, especially coliforms, and a reduced abundance of beneficial bacteria, such as lactobacilli and bifidobacteria (12).

Diet has a dominant role in shaping the gut microbiota, therefore the type of feeding in newborns has a certain impact on the assessment of the intestinal microbial groups. Remarkable differences were shown by Lee et al. (13): Actinobacteria was the predominant phylum in breastfed newborns, followed by Firmicutes and Proteobacteria; in contrast, in formula-fed infants, the proportions of Actinobacteria and Firmicutes were similar, followed by Proteobacteria. In addition, the gut microbiota of formula-fed infants contains a significant amount of the genera *Escherichia, Veillonella, Enterococcus*, and *Enterobacter*, whereas the content of *Lactobacillus* was low. The same work reports that the main genus in both breast- and formula-fed infants is *Bifidobacterium*, but the proportion resulted significantly higher in breastfed infants. The study of Mazzola et al. (14) also showed a reduced *Bifidobacterium* spp. count in mixed-fed infants (fed with at least 50% formula milk) with respect to breastfed. On the contrary, a differential representation of the genus *Bifidobacterium* was not detected in breastfed infants compared to formula-fed, although differences in the gut microbiota were observed in the two groups (15). Moreover, these studies detected lower bacterial richness and diversity in breastfed, probably for the presence of unique oligosaccharides in breast milk, which serve as selective metabolic substrates for a limited number of gut microbes (16).

Feeding type, influencing the composition of intestinal microbiota, could play a significant role in the pathogenesis of infant colics although after the first year of life these differences are lost (17). A recent study focused on colicky and non-colicky formula-fed infants, performed using FISH as bacterial counting technique, revealed a lower concentration of total bacteria and a higher abundance of Enterobacteriaceae in colicky formula-fed infants (18).

Several studies support the use of probiotics as therapeutic or preventive agents against various diseases, in particular enteric disorders but also human pathology which are not apparently linked to the microbial gut composition, such as allergies and autoimmune diseases (19, 20). A treatment with probiotics, whose beneficial effects on the gut microbiota disorders and on human health are well known, may have a protective effect from gastrointestinal disorders including colics and reduce the symptoms associated, leading to a correct microbial colonization in early infancy, when the gut microbiota is still in a period of adjustment.

Many studies have focused on the administration of *Lactobacillus reuteri* DSM 17938 as probiotic for the prevention or reduction of symptoms of functional gastrointestinal disorders, including colic, regurgitation, vomit and constipation with successful results (6, 21, 22). In particular, Savino et al. (23) evidenced a lower number of anaerobic gram negative bacteria, enterobacteriaceae and enterococci in colicky newborns that received *L. reuteri* compared to non treated babies. However, other *Lactobacillus* species, such as *L. delbrueckii* subsp. *delbruekii* DSM 20074 and *L. plantarum* MB 456, have shown inhibitory activity against gas-forming coliforms and they have the potential of being used in the management of infant colic (24). Differently, the administration of bifidobacteria for the treatment of these intestinal disorders remains scarcely investigated, although their role in the healthy newborn gut microbiota has been demonstrated as reviewed by Di Gioia et al. (25). A previous *in vitro* study described the capability of some strains belonging to *Bifidobacterium* genus, including *Bifidobacterium breve* strains, of inhibiting *in vitro* the growth of pathogens typical of the infant gastrointestinal tract including coliforms isolated from colicky newborns (26). Other studies demonstrated the efficacy of *B. breve*, strains for the treatment of different infant diseases: Li et al. (27) showed the usefulness in promoting the colonization of *B. breve* and the formation of a normal intestinal biota in low birth weight infants, Wada et al. (28) described beneficial effects of this species in immunocompromised pediatric patients on chemotherapy. Moreover, recent studies have evidenced the effectiveness of *B. breve* to reduce the risk of necrotizing enterocolitis in preterm infants (29, 30). In addition, the two strains *B. breve* B632 and BR03 have been investigated for their capability of colonizing human intestine, stimulating the immune response, competing against pathogens and their safety assessments have been also demonstrated (26, 31, 32). A recent study also showed the capability of these *B. breve* strains, used as probiotic for children with celiac disease, to act as a “trigger” element for the increase of other beneficial bacterial genus or phylum, like Firmicutes (33).

The aim of this study was to describe the effectiveness of a *B. breve* based probiotic formulation administered both to breast-fed and bottle-fed newborns in: 1) shifting the counts of targeted fecal microbial groups; 2) preventing colic symptoms and functional gastrointestinal disorders in a cohort of healthy newborns.

## Materials and methods

### Study design and samples collection

**Figure 1:**
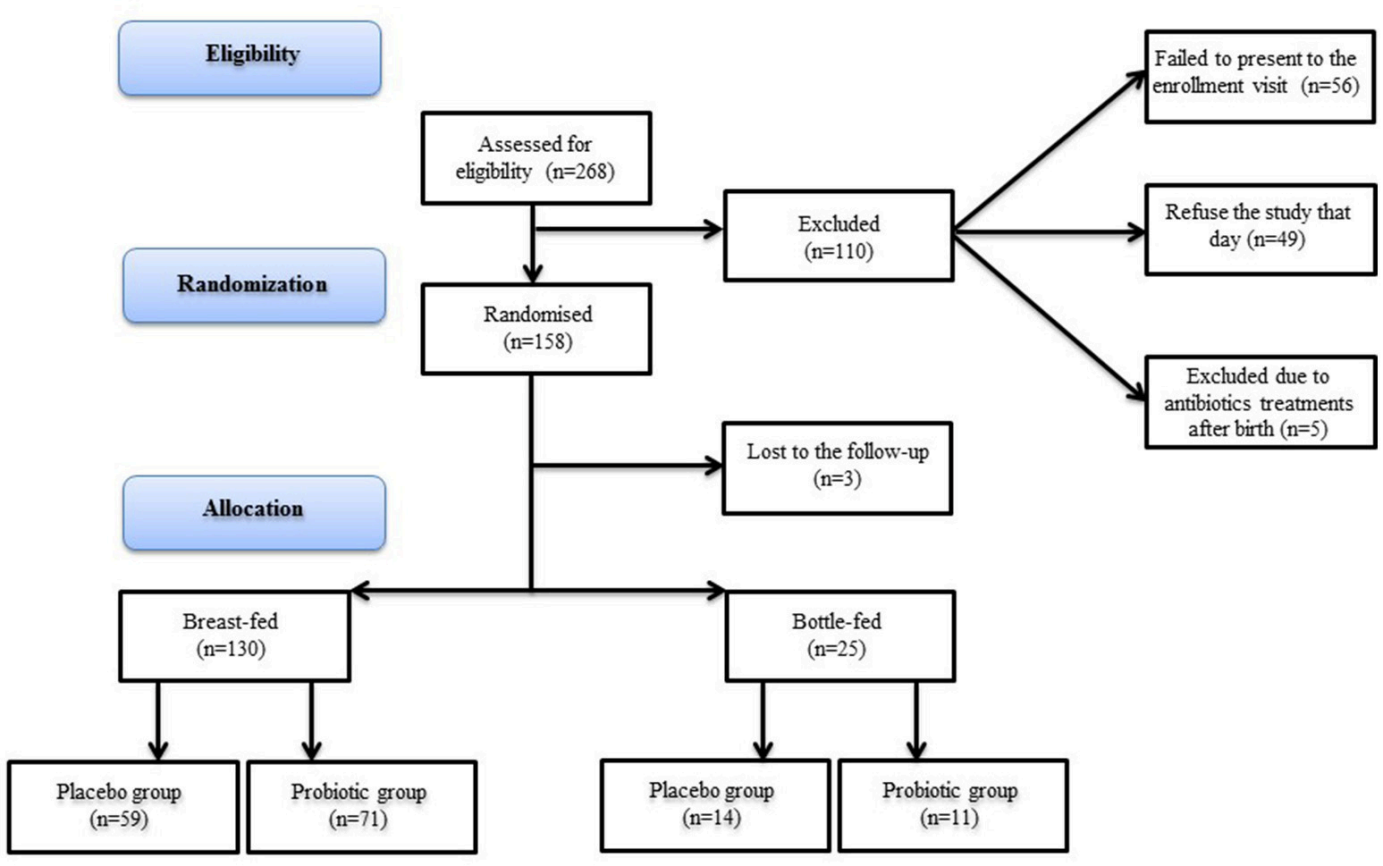
Study flow diagram.

This was a double-blind, randomized, placebo-controlled clinical trial (NCT03219931) approved by the Ethical Committee of the Maggiore della Carità Hospital (CE 63/13). The newborns were enrolled at the Department of Medical Sciences, Division of Pediatrics, University of Piemonte Orientale “A. Avogadro” in a period from November 2013 to September 2016. Newborns were recruited at birth and enrolled within 15 days from birth during the first visit (T0). Informed consent was obtained by parents at the enrolment, in accordance with the local Ethics Committee and Helsinki criteria. Patients were asked to perform a second visit (T1) after 90 days of treatment. The number of newborns assessed for eligibility [268], randomized [155], and allocated to the placebo or probiotic group is shown in Figure 1. They were recruited if healthy within 15 days from birth and born adequate for gestational age. Exclusion criteria were: 1) twin neonates; 2) treatments with any type of drug within the enrolment; 3) treatments with probiotics; 4) smoking mothers; 5) family history for congenital diseases; 6) history of prolonged jaundice. No specific dietary restrictions during lactation were recommended to the mothers, with the exception of other products containing probiotics. Patients were randomized using a computer-generated allocation sequence in Placebo or Probiotic group (1:1). The study personnel and parents were masked to the study group allocation. The original idea of the study was to recruit an equal number of breast-fed and bottle-fed newborns but, considering the difficulties in the enrolment in bottle-fed ones, we decided to go on with a different number of newborns belonging to the two groups (Figure 1).

The Probiotic group received a commercial probiotic formulation Bifibaby® (Probiotical S.p.A., Novara, Italy) containing *B. breve* for 90 days (T1) and the Placebo group received a placebo formulation for the same period. Probiotic formulation was a 1:1 mixture of 2 strains, *B. breve* BR03 (DSM 16604) and *B. breve* B632 (DSM 24706) prepared in an oily suspension, administered in a daily dosage of 5 drops containing 10^8^ CFU of each strain. Placebo was prepared with the same excipients without probiotic strains using an identical form of package.

### Clinical monitoring

Delivery and birth data were collected during the first visit. Anthropometric data (weight, height, head circumference) and type of feeding information were collected at both the first (T0) and second visit (T1).

Parents were asked to record on a daily diary minutes of inconsolable crying according to a validated questionnaire (41). They also recorded daily number of regurgitations, vomits, and evacuations, and colour and consistency of stools. The Bristol Stool Form Scale for children was given to parents (42). Colic was diagnosed according to the Rome IV consensus group (3).

Parents were also asked to report any adverse event (in particular constipation, vomit, allergic reactions, illness), treatments, number and type of infections, or abdominal pain occurred during the trial period. The adherence was monitored by biweekly phone calls, counting empty vials, and checking daily dairies.

### Stool samples collection

Faecal samples of newborns were collected twice, on enrolment (T0) and at the end of the intervention with probiotic/placebo (T1). The analyzed groups were therefore: Probiotic T0, Placebo T0, Probiotic T1, Placebo T1. Faecal samples were frozen immediately after collection at −80°C, in numbered screw-capped plastic containers, until they were processed for DNA extraction. Researchers performing DNA extraction and molecular analyses (qPCR) were blind to the group identity of patients (Probiotic or Placebo group).

### DNA extraction from faecal samples

DNA was extracted from 200 mg of faeces (preserved at −80°C after collection) using the QIAamp DNA Stool Mini Kit (Qiagen, West Sussex, UK) with a slight modification of the standard protocol: a supplementary incubation at 95°C for 10 min of the stool sample with the lysis buffer was added to enhance the bacterial cell rupture (43). Extracted DNA was stored at −80°C. The purity of DNA was determined by measuring the ratio of the absorbance at 260 and 280 nm (Infinite®200 PRO NanoQuant, Tecan, Mannedorf, Switzerland) and the concentration was evaluated by Qubit® 3.0 Fluorometer (Invitrogen, Life Technologies, CA, USA).

### Absolute quantification of selected microbial groups using quantitative PCR (qPCR)

Quantification of selected microbial groups or species usually monitored in studies related to infants (38, 43), i.e., *Bidobacterium* spp., *Lactobacillus* spp., *Bacteroides fragilis* group (comprising the most abundant species in human *B. fragilis, B. distasonis, B. ovatus, B. thetaiotaomicron, B. vulgatus*), *B. breve, Clostridium difficile, Escherichia coli*, and total enterobacteria, was performed with real-time PCR on DNA extracted from stool samples. The assays were carried out with a 20 μL PCR amplification mixture containing 10 μL of Fast SYBR® Green Master Mix (Applied Biosystems, Foster city, CA, USA) optimized concentrations of primers (Tables 1, 2), molecular grade H_2_O and 2 μL DNA obtained from faecal samples at a concentration of 2.5 ng/μL. *B. breve* analysis was performed using a TaqMan assay containing 12.5 μL of Universal TaqMan master mix (Applied Biosystems, Foster city, CA, USA) 300 nM of each primers and 100 nM of probe labeled with the 5′ reporter dye 6-carboxyfluorescein and the 3′ quencher NFQ-MGB (Applied Biosystems, Nieuwerkerk a/d IJssel, The Netherlands). The number of PCR cycles was 40.

**Table 1:** Primer sequences and qPCR conditions used in the different assays.

**Microorganism target**	**Primer**	**Sequence (5′-3′)**	**Amplicon length (bp)**	**References**
*Escherichia coli*	Eco-F	GTTAATACCTTTGCTCATTGA	340	(34)
	Eco-R	ACCAGGGTATCTAATCCTGTT		
*C. difficile*	Cdiff-F	TTGAGCGATTTACTTCGGTAAAGA	114	(35)
	Cdiff-R	TGTACTGGCTCACCTTTGATATTCA		
*Bifidobacterium* spp.	Bif-F	TCGCGTCYGGTGTGAAAG	243	(36)
	Bif-R	CCACATCCAGCRTCCAC		
*Lactobacillus* spp.	Lac-F	GCAGCAGTAGGGAATCTTCCA	349	(37)
	Lac-R	GCATTYCACCGCTACACATG		
*Bacteroides fragilis* group	Bfra-F	CGGAGGATCCGAGCGTTA	92	(38)
	Bfra-R	CCGCAAACTTTCACAACTGACTTA		
*B. breve*	F _IS	GTGGTGGCTTGAGAACTGGAT AG	118	(39)
	R_IS	CAAAACGATCGAAACAAACACTAAA		
	P_IS	TGATTCCTCGTTCTTGCTGT		
Enterobacteria	Ent-F	ATGGCTGTCGTCAGCTCGT	385	(40)
	Ent-R	CCTACTTCTTTTGCAACCCACTC		

**Table 2:** qPCR amplification protocols and primer concentrations.

**Target bacteria**	**Initial denaturation**	**Denaturation**	**Annealing**	**N. cycles**	**Fw (nM)**	**Rev (nM)**
***E. coli***						
Eco-F/Eco-R	95°C – 20 s	95°C – 3 s	60°C – 30 s	40	400	400
***C. difficile***						
Cdiff-F/Cdiff-R	95°C – 20 s	95°C – 3 s	60°C – 30 s	40	250	250
***Bifidobacterium*** **spp**.						
BifTOT-F/BifTOT-R	95°C – 20 s	95°C – 3 s	60°C – 35 s	40	200	300
***Lactobacillus*** **spp**.						
Lac-F/Lac-R	95°C – 20 s	95°C – 3 s	63.5°C – 30 s	40	200	200

The primer concentrations were optimized through primer optimization matrices in a 48-well plate and estimating the best Ct/ΔRn ratio. The different primers were also checked for their specificity utilizing the database similarity search program nucleotide-nucleotide BLAST (44). Moreover, to evaluate the specificity of amplification, analysis of product melting curve was performed after the last cycle of each amplification. The data obtained from the amplification were then converted to obtain the number of bacterial cells (Log CFU/g faeces) in accordance with the rRNA copy number available at the rRNA copy number database (45). Standard curves were constructed using 16S rRNA PCR products of type strains of each target microorganism; the standard microorganisms used were *B. breve* ReO2, *Lactobacillus plantarum* ATCC 14917, *B. fragilis* DSM 2151, *B. breve* B632 DSM 20213, *Clostridium sporogenes* ATCC 319, *E. coli* ATCC 8739. PCR products were purified with a commercial DNA purification system (NucleoSpin® Extract II kit, MACHEREY-NAGEL GmbH & Co. KG, Germany) and the concentration measured spectrophotometrically at 260 nm. Serial dilutions were performed and 10^2^, 10^3^, 10^4^, 10^5^, 10^6^, 10^7^ copies of the gene per reaction were used for calibration. Sample reactions were conducted in triplicate, with a negative control per each reaction.

### Statistical analysis

Data were expressed as mean ± SD. Skewed variables were log transformed. Daily data were divided in 9 categories representing the mean of 10 consecutive days (from 0 to 90 day).

According to the primary outcome, a sample of 58 individuals per group has been estimated to be sufficient to demonstrate a difference between placebo and probiotics of 0.70 Log CFU/g of bifidobacteria with a SD of 1.6, a 90% power, and a significance level of 95%, and a drop-out rate of 20% according to published data already available during the protocol design (32). According to the secondary outcome, a sample of 55 individuals per group has been evaluated sufficient to reduce of 30% the proportion of gastrointestinal disorders (colic, regurgitation, vomit, constipation) with an estimated prevalence of 40%, according to literature (1, 6).

Data of microbial counts were subjected to Shapiro test and Bartlett test in order to verify the normal distribution of data and homogeneity of variances. The baseline characteristics were compared with a Fisher's exact test for categorical variables and a two sample *t*-test or the Welch's *t*-test when appropriate for continuous variables. A two-way repeated measure ANOVA was performed to evaluate the time effect, the treatment effect and the interaction effects (model 1) on the dependent variables (minutes of crying, stool characteristics, episodes of vomits and regurgitation, microbial counts). Sum of squares type III was used. The following covariates were also subsequently introduced: sex, type of delivery (vaginal, caesarean, operative), intrapartum antibiotic prophylaxis (IAP), gestational age, neonatal weight (model 2). Model 3 also included the type of feeding during the 90 days (breast-, bottle-, mixed-feeding). Furthermore, in model 2 and 3, weight, length, and head circumference were also corrected for the corresponding variable at birth. All the statistical analyses were performed using R Statistical Software and SPSS for Windows version 17.0 (SPSS Inc., Chicago, IL, USA).

## Results

### Baseline characteristics of enrolled newborns

At birth, 268 newborns were assessed for eligibility and their parents accepted the study. One hundred and ten did not enter the study because failed to present to the enrollment visit (46), refused the study that day (47) or were excluded due to antibiotic treatments after birth (5). The 158 subjects were assigned randomly to placebo or probiotic group. Three of them were lost at the follow-up and were excluded (Figure 1). Of the 155 newborns who entered in the protocol, 130 were breast-fed (59 placebo, 71 probiotics) and 25 were bottle-fed (14 placebo, 11 probiotics). Eighty-one were males, and 74 females. Moreover, 139 neonates were born by vaginal, 10 by cesarean, and 6 from operative delivery. All the enrolled mothers were healthy without suffering of chronic diseases. Fifteen mothers received IAP. Three mothers have an episode of flu during the study (2 subjects in the breastfeeding group and 1 subject in the formula feeding group). No mother was treated with antibiotics during lactation.

Table 3 represents clinical data and microbiological fecal counts at baseline in the two groups of allocation (placebo and probiotic neonates). Only *Lactobacillus* spp. counts were higher in the placebo group than in the probiotic group at baseline.

**Table 3:** Auxological characteristics of the whole cohort at baseline (T0) according to the allocation treatment.

	**Placebo**	**Probiotic**
Gender (M/F)	34/39	47/35
Gestational age (weeks)	39.1 ± 1.2	39.3 ± 1.0
Neonatal weight (g)	3307.9 ± 397.5	3298.5 ± 362.7
Length (cm)	50.1 ± 2.0	50.1 ± 1.7
Head circumference (cm)	34.2 ± 1.4	33.9 ± 1.1
Delivery (V/C/O)	63/7/3	76/3/3
Days of life	10.6 ± 1.9	10.4 ± 2.4
Breast-/Bottle-feeding	59/14	71/11
Daily crying (min)	25.5 ± 28.8	28.8 ± 37.7
Stool frequency	3.8 ± 1.8	4.1 ± 1.9
Regurgitation episodes	1.7 ± 1.8	1.6 ± 1.4
Vomit episodes	0.1 ± 0.2	0.2 ± 0.4

*Data are expressed as mean ± SD. C, cesarean; O, operative; V, vaginal*.

Because it is well-known that feeding modulates gut microbial composition as well as clinical presentation also in neonates, we investigated if breast- and bottle-fed babies were different at baseline. Supplementary Table 1 represents clinical data and microbiological fecal counts at baseline in the two groups (breast fed and bottle-fed neonates). Crying time (*p* < 0.05) and stool frequency were higher (*p* < 0.04) and regurgitation episodes were less frequent (*p* < 0.05) in breastfed infants. Total enterobacteria (*p* < 0.004), *E. coli* (*p* < 0.03), and *B. fragilis* group (*p* < 0.01) counts were lower in breastfed than in bottle-fed newborns, also when corrected for confounders (sex, gestational age, neonatal weight, type of delivery, IAP, and days of life at the entry date).

### Microbiological results in whole cohort after probiotic and placebo administration

Table 4 shows the average crude microbial count obtained from the two groups of samples: probiotic and placebo. This analysis showed a significant increase of *B. breve* counts after 3 months. The other microbial groups did not show any significant difference.

**Table 4:** Mean counts (Log CFU/g of faeces) of different microbial groups analyzed in stool samples of the whole cohort.

**Target**	**Probiotic T0**	**Probiotic T1**	**Placebo T0**	**Placebo T1**
*Bifidobacterium* spp.	7.00 ± 1.41	7.51 ± 0.88	6.88 ± 1.14	7.29 ± 1.06
*B. breve*	4.45 ± 1.85	6.40 ± 1.31^*^	4.54 ± 1.5	5.33 ± 1.5
Enterobacteria	6.54 ± 1.23	6.38 ± 1.14	6.02 ± 1.4	6.5 ± 1.02
*E. coli*	6.72 ± 1.93	7.2 ± 1.36	6.35 ± 2.07	7.42 ± 1.24
*Lactobacillus* spp.	6.56 ± 1.28	5.60 ± 1.23	6.22 ± 1.06	5.28 ± 1.48
*B. fragilis* group	7.44 ± 2.14	7.62 ± 1.97	6.79 ± 2.19	7.23 ± 2.00
*C. difficile*	2.66 ± 1.48	2.82 ± 1.50	2.60 ± 1.32	3.06 ± 1.58

* *Significant changes at t-test (p < 0.02)*.

Following this first evaluation and considering the different sample dimension of breast- and bottle-fed newborns as well as differences of baseline microbial counts in these two groups, an analysis separating breastfed from bottle-fed newborns was carried out.

### Data evaluation of probiotic treatment on breastfed newborns

At baseline, the placebo group had less stool frequency (*p* < 0.03) and lower enterobacteria counts than the probiotic one (*p* < 0.01), also when corrected for confounders (sex, gestational age, neonatal weight, type of delivery, and days of life at the entry date). The marginal means for stool frequency and enterobacteria counts are: 3.2 ± 0.4 vs. 4.3 ± 0.3 (*p* < 0.01), and 5.86 ± 0.36 vs. 6.01 ± 0.38 Log CFU/g, (*p* < 0.03), respectively.

The compliance to the treatment was high. All the parents answered to the phone calls. 98.7% of them brought back correctly the empty vials. No adverse events were reported. Three infants (1 in placebo and 2 in probiotic) received simethicone for several days (<30 days) due to regurgitations.

After 90 days, 100 (43 in placebo, 57 in probiotics) of the 130 neonates were still breastfed. In the remaining 30 infants, 7 were bottle-fed (5 in placebo, 2 in probiotic), and the other 23 were in mixed feeding (11 in placebo, 12 in probiotic).

#### Clinical data

Considering clinical data, minutes of crying (*p* < 0.005) decreased over time without an effect of treatment also in the corrected models.

The number of the evacuation decreased over time (*p* < 0.0001), with an effect of treatment in those born after 40 weeks of gestational age (*p* < 0.03). Stool consistency was more type 5 at the Bristol Stool Chart (*p* < 0.03) over time. Children born after 40 weeks of gestational age had more frequently type 6 at the Bristol Stool Chart in those in probiotics (*p* < 0.0001).

**Figure 2:**
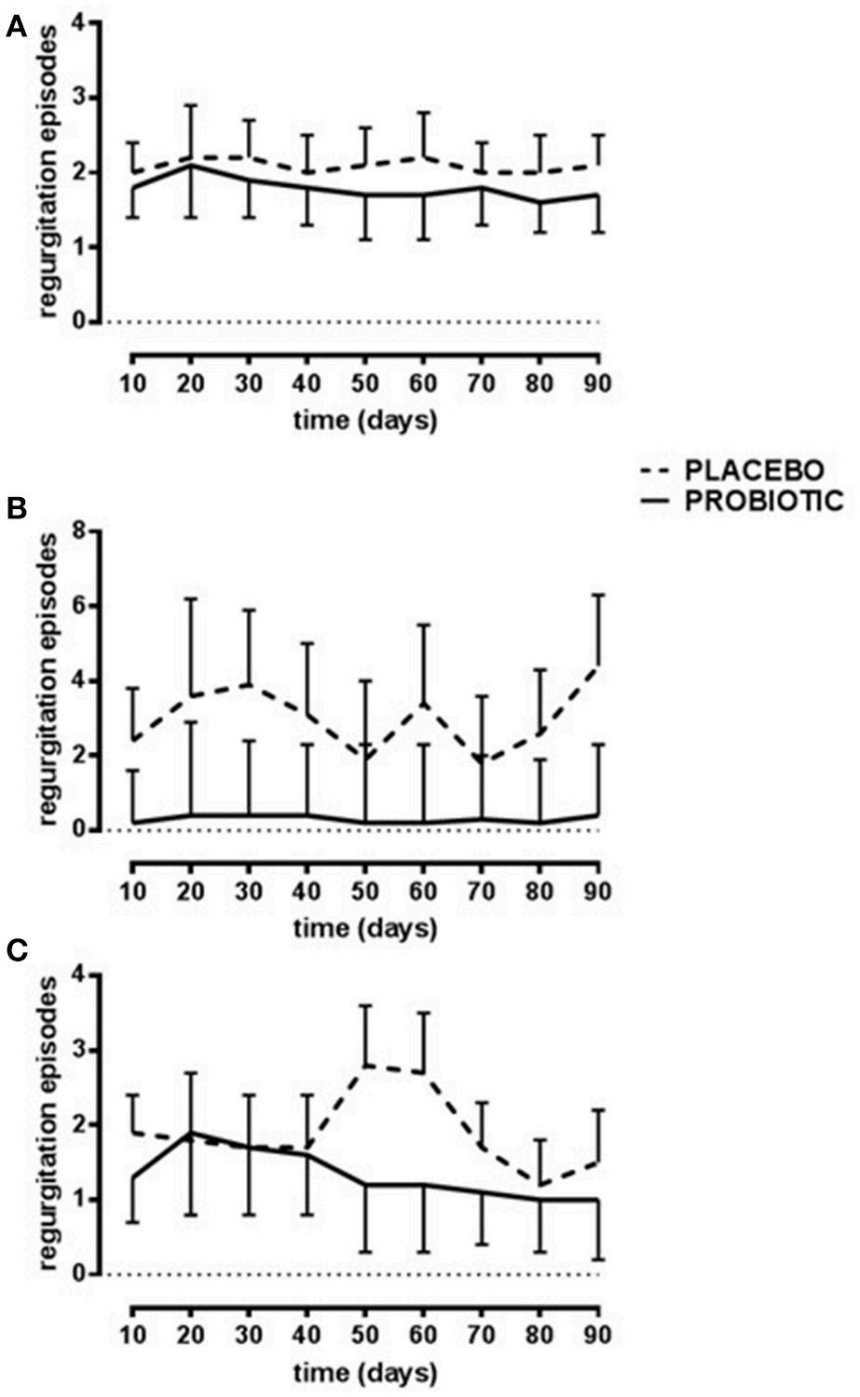
Number of daily regurgitations. Probiotic group (continuous line) and placebo group (dotted line). Breast-fed newborns **(A)**; Bottle-fed newborns **(B)**; Mixed-fed newborns **(C)**. Data are expressed as marginal mean ± SEM. Data are significant in interaction (*p* < 0.04; model 3). The residuals are not homogenous across the groups.

Episodes of regurgitation decreased constantly over time (*p* < 0.01) in the probiotic group diversely by placebo group accordingly to different type of feeding (*p* < 0.03, Figure 2).

Episodes of vomits decreased significantly with time in the probiotic group but not in the placebo group (*p* < 0.03). Moreover, during the 90 days the prevalence of colic infants was similar in the placebo (4 subjects, 6.8%) and probiotic group (6 subjects, 8.5%).

**Figure 3:**
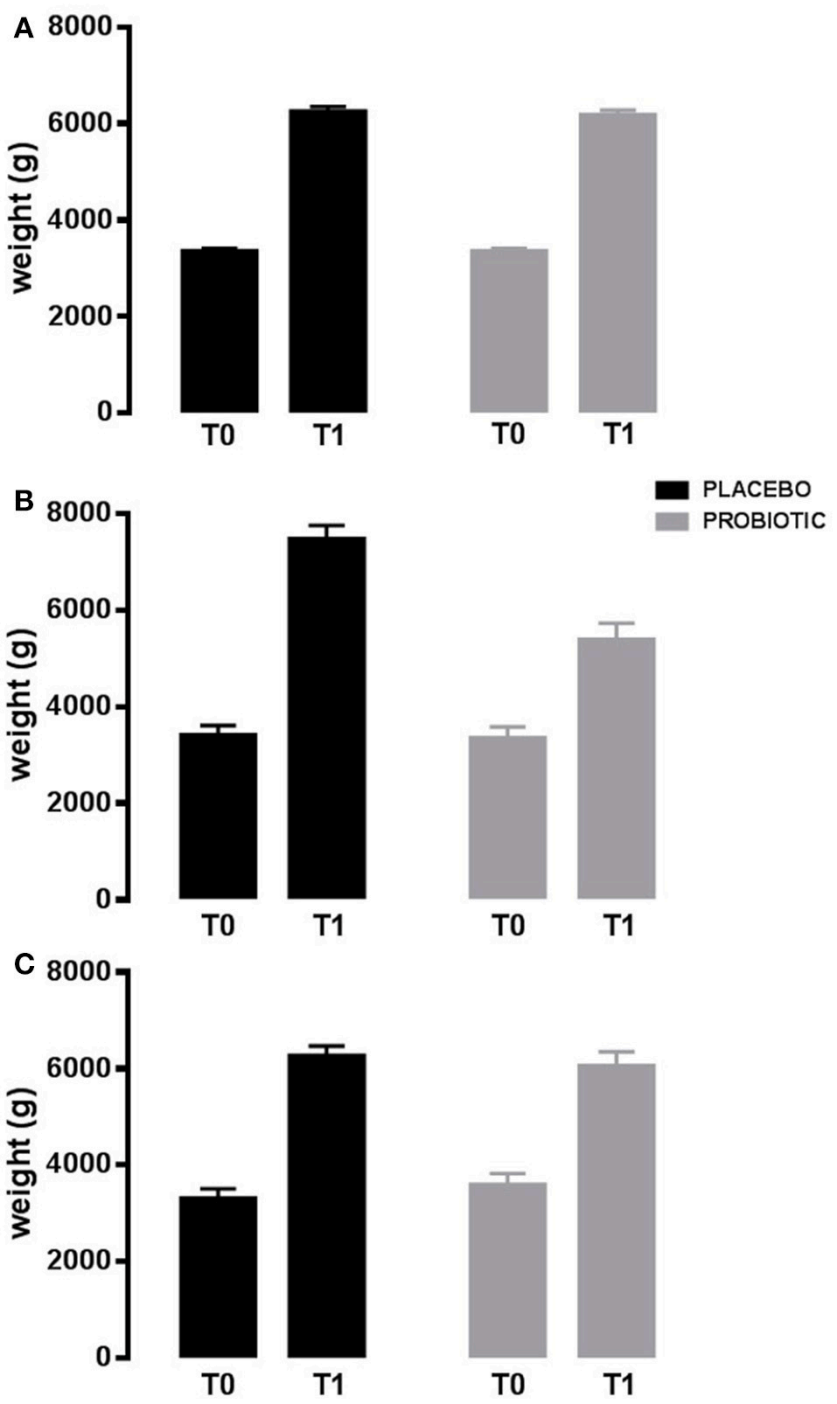
Weight variation in 90 days. Probiotic group (gray bar) and placebo group (black bar). Newborns born by vaginal delivery **(A)**; Newborns born by cesarean delivery **(B)**; Newborns born by operative delivery **(C)**. Data are expressed as marginal mean ± SEM. Data are significant in interaction (*p* < 0.03; model 2). T0: baseline. T1: after 90 days of placebo/probiotic.

Interestingly, also auxological variables were modified. Infants in probiotics had a lower increase in weight during the study in those born with a cesarean delivery (*p* < 0.03; Figure 3), and in those still breastfed or switched to bottle-fed during the study (*p* < 0.005). Diversely, infants in probiotics had a higher increase in head circumference in those bottle-fed or with a mixed-feeding (*p* < 0.01).

Table 5 describes marginal means of model 1. Supplementary Table 2 describes also corrected models.

**Table 5:** Clinical and anthropometric variations in the breast-fed group obtained with multivariable analysis of repeated measure.

**Target**	**Probiotic T0**	**Probiotic T1**	**Placebo T0**	**Placebo T1**
Crying(min)	25.4 ± 4.5	19.3 ± 2.9	32.1 ± 5.6	20.5 ± 3.7
Stool frequency	4.3 ± 0.2	2.1 ± 0.1	3.9 ± 0.2	2.0 ± 0.1
Stool color	6.0 ± 0.1	5.7 ± 0.1	6.0 ± 0.1	6.0 ± 0.1
Stool consistency	5.8 ± 0.1	5.7 ± 0.1	6.0 ± 0.1	5.6 ± 0.1
Regurgitations	2.0 ± 0.2	1.6 ± 0.2	1.8 ± 0.2	2.0 ± 0.3
Vomits	0.25 ± 0.05	0.11 ± 0.06^*^	0.10 ± 0.05	0.20 ± 0.07
Weight (g)	3310.9 ± 43.7	3465.6 ± 57.4	3321.5 ± 48.0	3452.2 ± 62.9
Length (cm)	50.1 ± 0.2	51.5 ± 0.2	50.2 ± 0.2	51.5 ± 0.2
HC (cm)	33.9 ± 0.1	34.9 ± 0.1	34.3 ± 0.1	35.0 ± 0.1

*All values are expressed as marginal means ± standard error. HC, head circumference*.

* Significance in interaction (time * treatment) effect (p < 0.03).

#### Microbial data

Total Enterobacteria (*p* < 0.005), *Bifidobacterium* spp. (*p* < 0.001) and *E. coli* (*p* < 0.001) changed within time, but the significance was lost when corrected for confounders. *C. difficile* did not change.

In those treated with probiotics, *B. fragilis* group members decreased within time in those born vaginally, whereas increased in the other infants (*p* < 0.04). Moreover, *B. breve* increased within time in those treated with the probiotics (*p* < 0.04). Microbiological data are reported in Table 6.

**Table 6:** Mean counts (Log CFU/g of faeces) of different microbial groups analyzed in stool samples of breast-fed newborns.

**Target**	**Probiotic T0**	**Probiotic T1**	**Placebo T0**	**Placebo T1**
*Bifidobacterium* spp.	7.11 ± 0.38	7.91 ± 0.27	6.59 ± 0.34	7.78 ± 0.24
*B. breve*	4.64 ± 0.50	6.10 ± 0.47^*^	4.40 ± 0.48	5.86 ± 0.46
Enterobacteria	6.01 ± 0.38	6.55 ± 0.35	5.86 ± 0.36	6.46 ± 0.34
*E. coli*	6.70 ± 0.57	7.46 ± 0.51	6.40 ± 0.54	7.06 ± 0.48
*Lactobacillus* spp.	6.22 ± 0.33	5.55 ± 0.33	6.28 ± 0.29	6.27 ± 0.35
*B. fragilis* group	6.34 ± 0.64	7.33 ± 0.61	6.31 ± 0.61	6.45 ± 0.58
*C. difficile*	2.70 ± 0.42	2.89 ± 0.48	2.82 ± 0.37	3.01 ± 0.42

*All values are expressed as marginal means ± standard error*.

* Significance in interaction (time * treatment) effect (p < 0.04).

### Data evaluation of probiotic treatment on bottle-fed newborns

At baseline, placebo group had less *Lactobacillus* spp. counts than probiotic one (*p* < 0.008). When corrected for confounders (sex, gestational age, neonatal weight, and days of life at the entry date), the statistical significance was lost, whereas *B. fragilis* group counts were higher in the probiotic group (marginal means are 6.62 ± 0.53 vs. 8.62 ± 0.67 Log CFU/g, *p* < 0.02).

#### Clinical data

Considering clinical data in both crude and corrected analysis, no changes were detected in minutes of crying, stool frequency and consistency, episodes of vomits or regurgitations. No infants had colic in both groups. Weight (*p* < 0.0006), length (*p* < 0.01), and head circumference (*p* < 0.005) increased with time without an effect of the treatment.

#### Microbial data

Total enterobacteria and *E. coli* did not change over time. *Bifidobacterium* spp. (*p* < 0.02) and *C. difficile* increased (*p* < 0.04) with time without a treatment's effect. In those treated with probiotics, *B. fragilis* group (*p* < 0.03) decreased and *B. breve* increased (*p* < 0.03), respectively with time. Microbiological data are reported in Table 7.

**Table 7:** Mean counts (Log CFU/g of faces) of different microbial groups analyzed in stool samples of bottle-fed newborns.

**Target**	**Probiotic T0**	**Probiotic T1**	**Placebo T0**	**Placebo T1**
*Bifidobacterium* spp.	6.80 ± 0.36	7.56 ± 0.36	6.79 ± 0.30	7.35 ± 0.30
*B. breve*	4.20 ± 0.35	6.42 ± 0.37^*^	4.11 ± 0.31	5.09 ± 0.33
Enterobacteria	7.30 ± 0.41	6.97 ± 0.34	6.75 ± 0.36	7.14 ± 0.30
*E. coli*	7.91 ± 0.95	7.38 ± 0.62	6.67 ± 0.66	7.51 ± 0.54
*Lactobacillus* spp.	6.81 ± 0.29	6.00 ± 0.35	6.05 ± 0.26	6.39 ± 0.31
*B. fragilis* group	8.65 ± 0.61	7.98 ± 1.84^*^	7.56 ± 0.56	8.33 ± 0.51
*C. difficile*	2.54 ± 0.44	3.16 ± 0.52	2.59 ± 0.39	3.35 ± 0.46

*All values are expressed as marginal means ± standard error*.

* Significance in interaction (time * treatment) effect (p < 0.03).

## Discussion

The use of bifidobacteria as probiotics in infants is established for some enteric diseases, the most common of which is diarrhea (25). However, although *in vitro* studies support the use of bifidobacteria against gas-forming coliforms (26), no clinical trials have been performed up to now on their use against infant colics. This work was focused on the evaluation of the effects on functional gastrointestinal symptoms, including colics, of integration of the infant diet with a *B. breve* based probiotic formulation.

The study has clearly shown the capability of the administered *B. breve* strains to survive to the gastric transit and to reach the neonatal intestine. In fact, although *B. breve* was detected in all fecal samples, a significant increase was shown upon strain administration. In agreement with Lee et al. (13), a reduction of *Lactobacillus* counts was observed in all groups of newborns over time and this is particularly evident in the probiotic treated group. This could be related to a high ability of *Bifidobacterium* spp. to influence gut microbiota composition, by enhancing the blooming of some species and reducing others, as observed in other studies regarding *Bifidobacterium* administration (25).

Feeding type is known to have a crucial role in shaping the infant intestinal microbiota (25, 48). Our study shows that, at the enrolment, when 7–15 days of breast- or bottle-feeding had already been done, some differences were present in the groups with different feeding type: total enterobacteria and *E. coli* counts were higher in bottle-fed than in breast-fed newborns, also when corrected for confounders. In addition, higher counts of *B. fragilis* were found in bottle-fed infants at the baseline, in agreement with the higher risk of infection generally observed in non breast-fed infants (49). This higher count was also evident after treatment, both in the probiotic and the placebo group, confirming the absolute importance of the starting feeding type in shaping the gut microbiota and, in particular, in reducing gram-negative bacteria amount. However, in bottle-fed infants, the mean counts of *B. fragilis* were higher at the end of the treatment in the placebo group with respect to the probiotic one, thus indicating a possible positive effect of the *B. breve* administration at least before weaning. The increase of *B. breve* is also observed in breast-fed newborns not treated with probiotics and this, as already mentioned before, once more highlights the positive role of breast milk in shaping the bifidobacteria community, also considering that *B. breve* is one of the most abundant species in the newborn gut (50). This increase is also supported by the presence of peptides and oligosaccharides in the human milk that provide the stimulation of the growth of bifidobacteria (47).

In addition to the microbial data, this study aims at monitoring the typical gastrointestinal symptoms of colics, i.e., regurgitation, vomit and constipation, all of them difficult-to-handle problems for caregivers. Results obtained from the applied models showed a decreased number of evacuations and an enhancement of stool consistency in breast-fed newborns after 90 days of probiotics. In addition, bottle-fed newborns showed an improvement of stool color. These data suggested an amelioration in the gastrointestinal transit which can be attributed to the probiotic intake. Moreover, the number of regurgitations and episodes of vomit was reduced after probiotic treatment. Similar results have already been demonstrated with a supplementation of *L. reuteri* (6). The reduction of these symptoms is particularly important because they also reduce parental anxiety and related consequences.

The reduction in regurgitation and vomit was not shown in the group of bottle-fed newborns. This result can be affected by the small size of the bottle-fed group of newborns. The study was not designed to evaluate differences between the two feeding regimens and authors are aware that the bottle-fed group was underpowered to reach the clinical outcomes. However, data related to the different feeding should be analyzed separately due to the unexpected significant differences in microbial composition at baseline. On the other hand, those breast-fed at the recruitment who switched to bottle-or mixed-feeding had an improvement with reduction of regurgitation episodes. This is an important achievement also considering that the number of newborns bottle-fed since the beginning of life is generally low as, usually, a starting feeding with mother milk is applied (51).

In this study, daily infant crying time did not show any difference between probiotic and placebo groups in spite of the improvement of the gastric transit due to the probiotic administration. This result is contrasting with other reports in literature. Several causes could be considered, first of all the inaccuracy of the count of minutes of crying through self-report diaries, although validated, in particular for such a prolonged time. Analysis of any existing tool to monitor daily crying have been demonstrated to be inaccurate, difficult or not validated for a prolonged time of observation (52). Moreover, we evaluated the effects over 3 months, whereas the majority of the studies are related to probiotic treatment no longer than 4 weeks (1, 53). Moreover, other confounding factors may have a role after the second months of life, in particular, if we consider the efficacy on the other gastro-intestinal parameters. Furthermore, the prevalence of colic infants was similar but very low in both placebo and probiotic groups. This is a consequence of considering in the study healthy newborns. Studies including only colic infants are needed in the future.

The main unexpected and interesting result of the study was related to the auxological parameters. Clinical trials on the effect of probiotics on neonatal growth parameters are scarce. In our population, infants born by cesarean section had a lower catch-up growth in weight if treated with the probiotic. This result is of crucial interest in planning further intervention studies. Gut colonization by environmental microorganisms occurs during or immediately after the birth, whereas, in infants delivered by cesarean section, gut colonization is delayed and often altered, in particular modifying *Bifidobacterium* and *Lactobacillus* counts (25). Increasing epidemiological data suggested that children born by cesarean section have an increased risk to develop obesity later in life (54, 55). How the genetic background and the environment affect mechanisms that control appetite, weight regulation and metabolic disorders linked to overweight, and the immune education is poorly understood. Gestation, delivery, postnatal nutrition (lactation and weaning) have been identified as critical periods to program the nutritional and hormonal control of the offspring. Some Authors suggest that the sudden modification of the initial conditions may disrupt the physiological process predisposing to certain diseases (46, 56, 57) and alterations in the precocious colonization have a role (54, 55). Our data suggest that a treatment with *B. breve* strains in the first 3 months of life is able to influence the microbiota composition and this is associated with a concomitant lower weight gain in the population at higher risk of metabolic disturbances in later life. Other authors failed to show changes in weight in neonates treated with other probiotics (53, 58). Differences should be secondary to the strains or, more probably, to the timing of the treatment being our protocol designed on 3 months, differently from the majority of the studies which followed infants for 1 month. In our study, the effect on weight was associated to an increase in head circumference. These data suggest that the probiotic treatment protects against a growth failure, as recently demonstrated for a multi-strain probiotic containing bifidobacteria in very low birth weight children exposed to antibiotics (59).

The main limitation of this study is related to the small sample size of the bottle-fed population. However, this is a consequence of the inclusion criteria (healthy neonates) in a condition in which breastfeeding must be the first choice (51). Furthermore, although we used a validated questionnaire for daily infant crying, the other questionnaires used to record other gastrointestinal symptoms are not validated. On the contrary, the strengths of our study are a treatment prolonged for more than 4 weeks, the inclusion of neonates not exposed to antibiotics, the evaluation of many confounders, in particular regarding birth and changes in feeding over time.

In conclusion, our study demonstrates that the administered *B. breve* strains can reach the intestine of healthy newborns, preventing functional gastrointestinal disorders and reducing the precocious weight gain, at least in the absence of antibiotic interferences. No adverse events were reported, suggesting the safety of the product in this regimen. Prospective longitudinal evaluations should be useful to further investigate if a precocious short treatment in this critical window has also advantages later in life.

## Author contributions

DD and GB conceived and designed the study. EG and AS were responsible for patients recruitment and collection of clinical data. IA and NB performed the qPCR experiments and contributed to the writing of the paper. FP performed the statistical analysis, interpreted the results, and contributed to the writing of the paper. DD, SB, LB, and FP critically revised and approved the final manuscript. LM and MP designed the probiotic supplement.

### Conflict of interest statement

Authors LM and MP were employed by the company Biolab Research Srl, Novara, Italy, performing all Research & Development activities for Probiotical SpA, Novara, Italy. The other authors declare that the research was conducted in the absence of any commercial or financial relationships that could be construed as a potential conflict of interest.
