# Supplementary material for: Three-Month Feeding Integration With Bifidobacterium Strains Prevents Gastrointestinal Symptoms in Healthy Newborns
Source: Front Nutr. 2018 May 25;5:39. doi: 10.3389/fnut.2018.00039 (PMC5980983; doi:10.3389/fnut.2018.00039)
Supplement: Supplementary file 1 [file Table_1.DOCX]

Supplementary Material

**Three-month Feeding Integration with *Bifidobacterium* Strains Prevents Gastrointestinal Symptoms in Healthy Newborns**

Irene Aloisio, Flavia Prodam , Enza Giglione, Nicole Bozzi Cionci, Arianna Solito, Simonetta Bellone, Loredana Baffoni, Luca Mogna, Marco Pane, Gianni Bona, Diana Di Gioia^*^

*** Correspondence:** Corresponding Author: diana.digioia@unibo.it

**Supplementary Table 1.** Auxological characteristics of the whole cohort at baseline (T0) according to the type of feeding.

|  | **Breastfed neonates** | **Bottle-fed neonates** |
| --- | --- | --- |
| Gender (M/F) | 64/66 | 17/8 |
| Gestational age (weeks) | 39.2±1.1 | 39.1±1.2 |
| Neonatal weight (g) | 3315.7±367.4 | 3236.4±432.7 |
| Length (cm) | 50.2±1.8 | 49.7±1.6 |
| Head circumference (cm) | 34.0±1.3 | 34.0±1.3 |
| Delivery (V/C/O) | 119/6/5 | 20/4/1 |
| Days of life | 10.5±2.2 | 10.6±2.2 |
| Daily crying (min)* | 29.2±36.3 | 19.6±18.6 |
| Stool frequency* | 4.1±1.8 | 3.4±1.8 |
| Regurgitation episodes* | 1.6±1.3 | 2.1±2.5 |
| Vomit episodes | 0.1±0.4 | 0.1±0.3 |
| *Bifidobacterium* spp(Log CFU/g) | 6.97±1.33 | 6.79±1.11 |
| *B. breve* (Log CFU/g) | 4.68±1.73 | 4.15±1.14 |
| Enterobacteria (Log CFU/g)* | 6.07±1.31 | 6.99±1.37 |
| *E. coli* (Log CFU/g)* | 6.15±1.85 | 7.21±1.41 |
| *Lactobacillus* spp (Log CFU/g) | 6.48±1.18 | 6.38±1.02 |
| *B. fragilis* group (Log CFU/g)* | 6.75±2.25 | 7.96±2.07 |
| *C. difficile* (Log CFU/g) | 2.58±1.45 | 2.57±1.45 |

Data are expressed as mean±SD. Abbreviations: C: cesarean; O: operative; V: vaginal.

*significant differences at both Welch’s t-test and ANCOVA (corrected for sex, gestational age, neonatal weight, type of delivery, and days of life at the entry)
