# Supplementary material for: Three-Month Feeding Integration With Bifidobacterium Strains Prevents Gastrointestinal Symptoms in Healthy Newborns
Source: Front Nutr. 2018 May 25;5:39. doi: 10.3389/fnut.2018.00039 (PMC5980983; doi:10.3389/fnut.2018.00039)
Supplement: Supplementary file 2 [file Table_2.DOCX]

Supplementary Material

**Three-month Feeding Integration with *Bifidobacterium* Strains Prevents Gastrointestinal Symptoms in Healthy Newborns**

Irene Aloisio, Flavia Prodam , Enza Giglione, Nicole Bozzi Cionci, Arianna Solito, Simonetta Bellone, Loredana Baffoni, Luca Mogna, Marco Pane, Gianni Bona, Diana Di Gioia^*^

*** Correspondence:** Corresponding Author: diana.digioia@unibo.it

**Supplementary Table 2.** Clinical and anthropometric variations obtained with multivariable analysis of repeated measure.

|  | **Model 1** | | | **Model 2** | | | **Model 3** | | |
| --- | --- | --- | --- | --- | --- | --- | --- | --- | --- |
|  | time | treatment | Interaction | time | treatment | Interaction | time | treatment | Interaction |
| Crying(min) | F:  4.218  p<  0.005 | F: 0.236 | F: 0.788 | F:0.635 | F: 1.330 | F: 1.330 | F:0.563 | F: 0.144 | F: 0.442 |
| Stool frequency | F: 60.722  p < 0.0001 | F: 0.334 | F: 0.699 | F:0.570 | F: 0.228 | F: 2.423  p=0.065^a^ | F:0.821 | F: 0.048 | F: 2.513  p<0.04^a^ |
| Stool color | F: 2.41  p < 0.03 | F: 0.082 | F: 1.323 | F:0.844 | F: 0.002 | F: 2.292  p<0.03^b^ | F:0.374 | F: 0.045 | F: 1.988  p<0.04^b^ |
| Stool consistency | F: 2.410  p<0.01 | F: 0.082 | F: 1.323 | F:1.035 | F: 1.375 | F: 4.308  p<0.001^c^ | F:2.210  p<0.01 | F: 0.516 | F: 3.983  p<0.001^c^ |
| Regurgitations | F: 2.212  p<0.01 | F: 0.062 | F: 1.792  P:0.076 | F:0.999 | F: 0.583 | F: 0.240 | F:0.983 | F: 0.096 | F: 2.000  p<0.04^d^ |
| Vomits | F: 1.035 | F: 0.477 | F: 3.061 p<0.03 | F:0.486 | F: 4.351  p<0.01 | F: 2.177  p<0.02^e^ | F:0.178 | F: 0.718 | F: 2.000  p<0.04^d^ |
| Weight (g) | F: 25.967  p<0.0001 | F: 0.001 | F: 0.183 | F:27.042  p<0.0001 | F: 3.431 | F: 3.428  p<0.03^e^ | F:30.478  p<0.0001 | F: 2.630 | F: 5.597  p<0.005^d^ |
| Length (cm) | F: 92.391  p<0.0001 | F: 0.065 | F: 0.005 | F:24.272  p<0.0001 | F: 0.248 | F: 1.106 | F:24.878  p<0.0001 | F: 0.089 | F: 0.356 |
| HC (cm) | F: 70.631  p<0.0001 | F: 1.601 | F: 2.399 | F:20.172  p<0.0001 | F: 0.030 | F: 0.137 | F:23.009  p<0.0001 | F: 0.002 | F: 4.532  p<0.01^e^ |

Abbreviation: HC, head circumference.

Model 1. A two-way repeated measure ANOVA was performed to evaluate the time effect, the treatment effect and the interaction effects on the dependent variables (minutes of crying, stool characteristics, episodes of vomits and regurgitation, clinical characteristics).

Model 2. Model 1 plus the following covariates: sex, type of delivery (vaginal, caesarean, operative), IAP, gestational age, neonatal weight

Model 3. Model 1 plus the following covariates: sex, type of delivery (vaginal, caesarean, operative), IAP, gestational age, neonatal weight, feeding during the 90 days (breast- bottle-, mixed-feeding).

Significant interactions in model 2 and 3: a: time*treatment*gestational age*sex; b: time*treatment*sex* type of delivery; c: time*treatment*gestational age; d: time*treatment*type of feeding at visit 2; e: time*treatment; e: time*treatment* type of delivery
